# Redefining therapeutic landscapes: clinicopathological insights into low and ultra-low HER2 expression in male breast cancer

**DOI:** 10.1186/s13000-025-01632-3

**Published:** 2025-04-15

**Authors:** Jiuyan Shang, Jiaxian Miao, Shuyao Niu, Xuemei Sun, Yueping Liu

**Affiliations:** Department of Pathology, The Fourth Hospital of Hebei Medical University, No. 12 Jiankang Road, Shijiazhuang, 050011 Hebei China

**Keywords:** Male breast cancer, Human epidermal growth factor receptor 2, Hormone receptor, Clinicopathological features, Survival

## Abstract

**Objective:**

With the emergence of new antibody coupled drugs, the treatment decisions of patients with low and ultra-low HER2 expression have been reshaped. However, the epidemiological characteristics of relatively rare male breast cancer are still unclear. This study discusses the clinicopathological and immunophenotypic characteristics of male invasive breast cancer with low and ultra-low HER2 expression.

**Methods:**

The clinicopathological and immunophenotypic features of 106 cases of male invasive breast cancer were retrospectively analyzed. HER2 was evaluated according to ASCO/CAP guidelines. The cutoff value of HER2 positive cell staining was > 10%. HER2 negative cases were divided into HER2 low expression (IHC = 1+/2 + and ISH without amplification) and HER2-0 (IHC-0, HER2 null and < 10% weak staining of cell membrane). The clinicopathological characteristics and prognosis of the cases were collected.

**Results:**

106 male patients with invasive breast cancer from 2015 to 2024 were included in this study, and more than 85% of male breast cancer histological types were invasive ductal carcinoma. Immunophenotype: There were 23 cases of HER2-zero (including 13 cases of HER2 ultra-low), 72 cases of HER2 low, 11 cases of HER2 positive, and the HER2 positive rate was 10.38%, and the incidence of low expression was 67.93%; The incidence of HER2 low in male breast cancer was significantly higher than that in female breast cancer, and the difference was statistically significant (*P* < 0.05). In terms of prognosis, there was no statistical difference between HER2 low male breast cancer and female breast cancer (*P* > 0.05). There was no statistical difference in survival prognosis between different HER2 status in the male breast cancer cohort.

**Conclusion:**

Male invasive breast cancer is rare, and it is more common in the elderly over 60 years old. The positive rate of ER and PR is high, and the incidence of HER2 low is high. The high HER2 low expression rate of male breast cancer can provide a new anti-HER2 treatment decision.

## Introduction

Male breast cancer (MBC) is relatively rare, accounting for approximately 1% of all diagnosed breast cancers [1–2]. With the increasing incidence of male breast cancer [3–4], the treatment and prognosis of this particular group has received increasing attention [5]. Currently, the treatment of male breast cancer is mainly based on the treatment of post-menopausal female patients [6]. Because male breast tissue is closer to the skin, MBC usually presents as locally advanced disease, often with regional lymphatic metastases. At the molecular level, BRCA2 mutations are a known risk factor for male breast cancer development. The results suggested that alterations in BRCA2 are very prevalent in MBC and raise the possibility of using PARP inhibitors in this population [7–8]. Prognostically, the overall risk of death in male breast cancer patients is approximately 43% higher than in female breast cancer patients [9].

Human epidermal growth factor receptor-2 (HER2) serves as an important biomarker for molecular typing and therapeutic decisions in breast cancer, although anti-HER2-targeted therapeutic agents can significantly improve survival of HER2-positive breast cancer patients, but HER2-positive rates in male breast cancer patients are not only lower than those in female breast cancer, but also more severe at diagnosis and have a worse prognosis [7]. With novel HER2 antibody-drug conjugate (ADC) drugs showing survival benefit in HER2-positive, low-expressing patients with advanced and inoperable locally advanced breast cancer, whether male breast cancer could benefit from ADC-based drugs deserves further investigation. This study explores the clinicopathological features of HER2 low-expressing male breast cancer and compares it with HER2-negative male breast cancer to assess its prognosis.

## Materials and methods

### Patients

Male patients with invasive breast cancer admitted to the Fourth Hospital of Hebei Medical University from January 2015 to June 2024 were screened and evaluated for HER2 according to the latest version of ASCO/CAP guidelines. We collected the detailed information about MBC patients, including their diagnosis date, patient and tumor characteristics, surgical and neo-/adjuvant treatments, recurrence events, cause and date of death, secondary cancers, and comorbidities. Immunohistochemical (IHC) staining for HER2 was performed in all cases and the results were counted.

The cut-off value for HER2-positive cell staining was > 10%. HER2-negative cases were classified as HER2 low expression, which were IHC = 1+/2 + and no amplification by in situ hybridization (ISH) and HER2-0 (IHC-0, where ultra-low is IHC-0 with faint membrane staining), and HE sections and HER2 immunohistochemical sections of all patients were reviewed by 2 attending and above pathologists in a double-blind method to refine the clinicopathological data. And 93 female patients diagnosed with invasive carcinoma of the breast at the same time were simply randomly selected as controls using the lottery method to collect and refine the pathological data of the control group.

### Methods

Retrospective analysis of male breast cancer patients who met the criteria and control female breast cancer patients, HER2 was detected by IHC as well as fluorescence in situ hybridization (FISH), where the immunohistochemical method was performed using Roche rabbit monoclonal primary antibody (clone number: 4B5) and detected using BenchMarK XT fully automated IHC instrument. Estrogen receptor (ER) and progestogen receptor (PR) were detected by immunohistochemical staining SP method, with positive and negative external controls in each batch. Clinicopathological data were collected from the whole cohort of patients to analyze the biological characteristics of HER2 low male breast cancer.

### Interpretation of hormone receptors and HER2

According to the ASCO/CAP guidelines [10], HER2 IHC staining results were determined, HER2 0: no staining is observed HER2-null or membrane staining that is incomplete and is faint/barely perceptible and in < 10% tumor cells; HER2 1+: incomplete membrane staining that is faint/barely perceptible and in > 10% of tumor cells; HER2 2+: weak to moderate complete membrane staining in > 10% of tumor cells; or circumferential membrane staining that is complete, intense, and in ≤ 10% of tumor cells; HER2 3+: circumferential membrane staining that is complete, intense, and in > 10% of tumor cells. For HER2 2 + cases, the ISH method was used for further testing, where HER2-zero was determined as HER2 negative; 1 + and 2 + with no ISH amplification as HER2-low, 2 + with ISH amplification and 3 + as HER2 positive. Hormone receptor (HR)-positive, at least 1% of infiltrating tumor cells showed immunostaining.

### Statistical analysis

Statistical software SPSS 24.00 was used for statistical analysis and processing. Non-normally distributed measures were described using percentile and median, and count and rank data were described using frequency and percentage. The χ2 test or Fisher’s exact probability method was used for comparison of count data, and the rank sum test was used for comparison of rank data; Kaplan-Meier curves were used to calculate survival rates, and Log-rank tests were used to compare survival differences. A two-sided test was used, and differences were considered statistically significant at *P* < 0.05.

## Results

### Population description and follow up

**Fig. 1:**
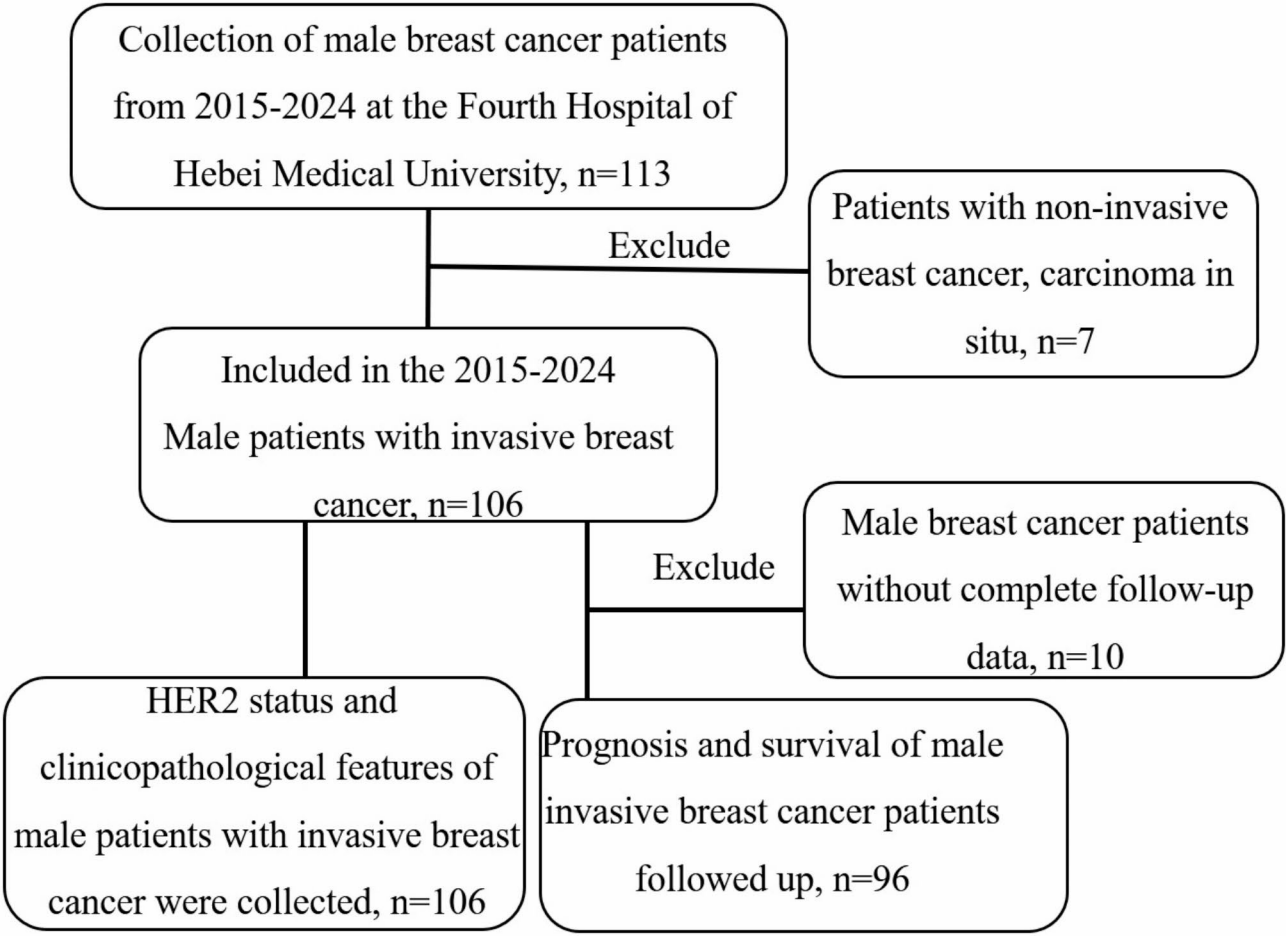
Flow chart of study population selection

**Fig. 2:**
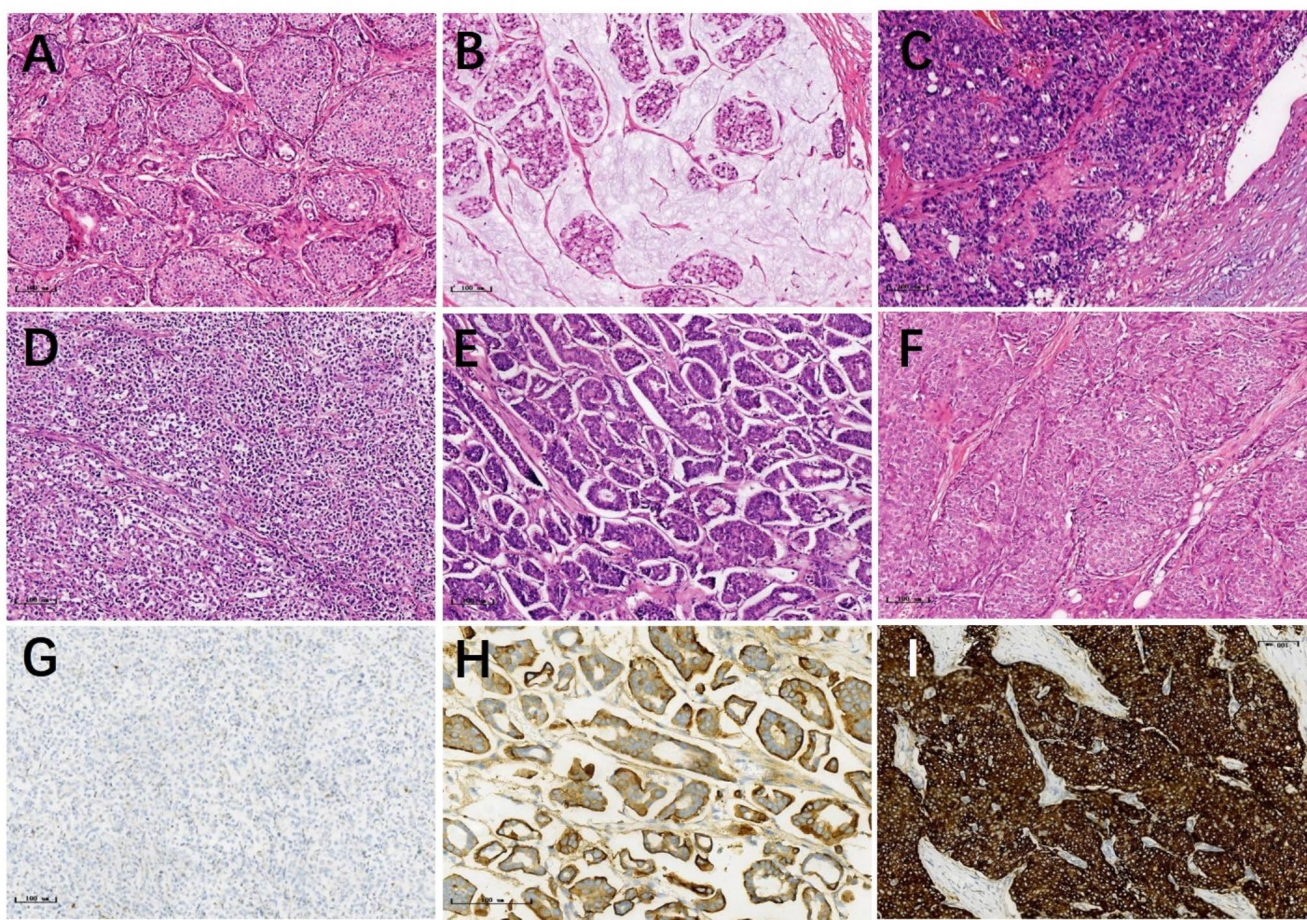
Histological types of male breast cancer; **A**, invasive carcinoma of no special type; **B**, mucinous carcinoma; **C**, encapsulated papillary carcinoma with invasive; **D**, invasive lobular carcinoma; **E**, invasive micropapillary carcinoma (IMPC); **F**, neuroendocrine carcinoma, G2; **G**, invasive lobular carcinoma E-cadherin staining, negative expression; **H**, immunohistochemical EMA staining, positive on the interstitial side around the micropapillary (reversed polarity); **I**, immunohistochemical Syn staining, strong positive expression

One hundred and six male patients with invasive breast carcinoma from 2015 to 2024 were included (Fig. 1), of whom the youngest was 37 years old, the oldest was 85 years old, and the median age was 65 years; 59 cases on the left side, 46 cases on the right side, and 1 case with a double breast mass, accounting for approximately 0.89% of breast cancer patients in the same period. There were 93 cases of invasive ductal carcinoma, including 5 cases with invasive micropapillary carcinoma component and 1 case with combined mucinous carcinoma component; other breast cancer subtypes included; 2 invasive cribriform carcinoma, 1 invasive lobular carcinoma, 2 mucinous carcinoma, 3 solid papillary carcinoma with invasive and mucus secretion, 2 neuroendocrine tumour (graded G2), 3 encapsulated papillary carcinoma of the breast with invasive (Fig. 2). Histological grading: 8 cases of Nottingham grade 1, 76 cases of grade 2, 12 cases of grade 3, and 10 cases of undetermined grade.

The control group included 93 female breast cancer patients in the same period, of which the minimum age was 27 years, the maximum age was 83 years, and the median age was 54 years; 51 cases on the left side, 41 cases on the right side, and 1 case of double breast mass. There were 69 cases of invasive ductal carcinoma of the breast, including 1 case combined with metaplastic carcinoma and 3 cases combined with invasive micropapillary carcinoma; 11 invasive lobular carcinoma and other rare breast cancer subtypes included: 1 invasive cribriform carcinoma, 2 carcinomas with apocrine differentiation, 3 neuroendocrine tumor (graded G2), 1 mucinous carcinoma with ductal carcinoma in situ, 1 malignant adenomyoepithelioma, 2 invasive solid papillary carcinoma, 1 metaplastic carcinoma, and two other patients had two masses (of which mass one was an invasive ductal carcinoma and mass two was an invasive lobular carcinoma). Histologic grading: 4 cases of Nottingham grade 1, 61 cases of grade 2, 25 cases of grade 3, and 3 cases of undetermined grade.

There were differences in age, HER2 status, ER, and PR status between male and female breast cancers (*P* < 0.05), with male breast cancers occurring more commonly above 60 years of age, and the rates of HER2 low expression, ER positivity, and PR positivity were higher than those of female patients (Table 1).

**Table 1:** Baseline characteristics of male and female breast cancer

Demographics	Total(*n* =199 )	Male(*n* =106 )	Female(*n* = 93)	χ2	*P* Value*
**Age**					
**<60 years**	81	28 (26.42%)	53 (56.99%)	19.186	0.000*
**≥ 60 years**	118	78 (73.58%)	40 (43.01%)		
**Laterality**					
**Right**	97	46 (43.40%)	51 (54.84%)	2.660	0.264
**Left**	100	59 (55.66%)	41 (44.09%)		
**Other**	2	1 (0.94%)	1 (1.08%)		
**Maximum tumor diameter**					
**<2**	52	32 (30.19%)	20 (21.51%)	4.117	0.128
**≥ 2**	139	72 (67.92%)	67 (72.04%)		
**Other**	8	2 (1.89%)	6 (6.45%)		
**Lymph node metastases**					
**Yes**	73	42 (39.62%)	31 (33.33%)	0.8448	0.358
**No**	126	64 (60.38%)	62 (66.67%)		
**HER2**					
**0**	48	23(21.69%)	25 (26.88%)	6.675	0.036*
**Low**	120	72 (67.93%)	48 (51.61%)		
**Positive**	31	11 (10.38%)	20 (21.51%)		
**HER2**					
**0**	48	23(21.70%)	25 (26.88%)	16.483	0.002*
**1+**	55	27 (24.47%)	28 (30.11%)		
**2 + ISH -**	65	45 (42.45%)	20 (21.51%)		
**2 + ISH +**	9	6(5.66%)	3 (3.23%)		
**3+**	22	5(4.72%)	17 (18.28%)		
**Estrogen Receptor**,** ER**					
**Negative**	27	7 (6.60%)	16 (17.20%)	5.446	0.026*
**Positive**	172	99 (93.40%)	77 (82.80%)		
**Progestogen Receptor**,** PR**					
**Negative**	32	8 (7.55%)	19 (20.43%)	7.011	0.008*
**Positive**	167	98(92.45%)	74 (79.57%)		
**Ki-67**					
**<14%**	34	19 (17.92%)	15 (16.13%)	0.113	0.737
**≥ 14%**	165	87 (82.08%)	78 (83.87%)		

### Distribution of HER2 low expression in male breast cancer cohort

**Fig. 3:**
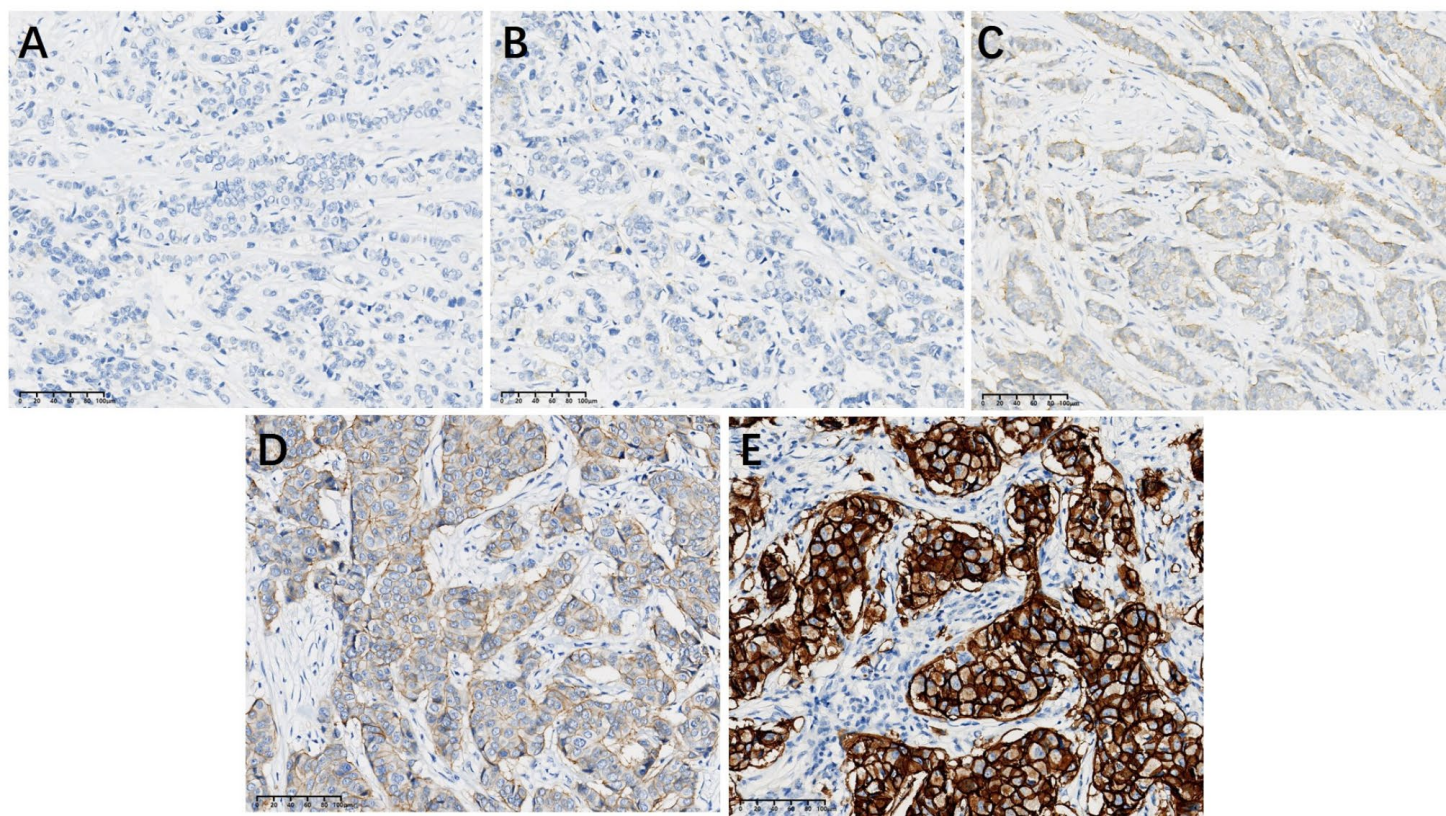
Immunohistochemical HER2 staining for male breast cancer; **A**, HER2-null (IHC no staining); **B**, HER2-ultralow(IHC < 10% weak staining of cell membrane); **C**, HER2 IHC 1+; **D**, HER2 IHC 2+; **E**, HER2 IHC 3+

**Fig. 4:**
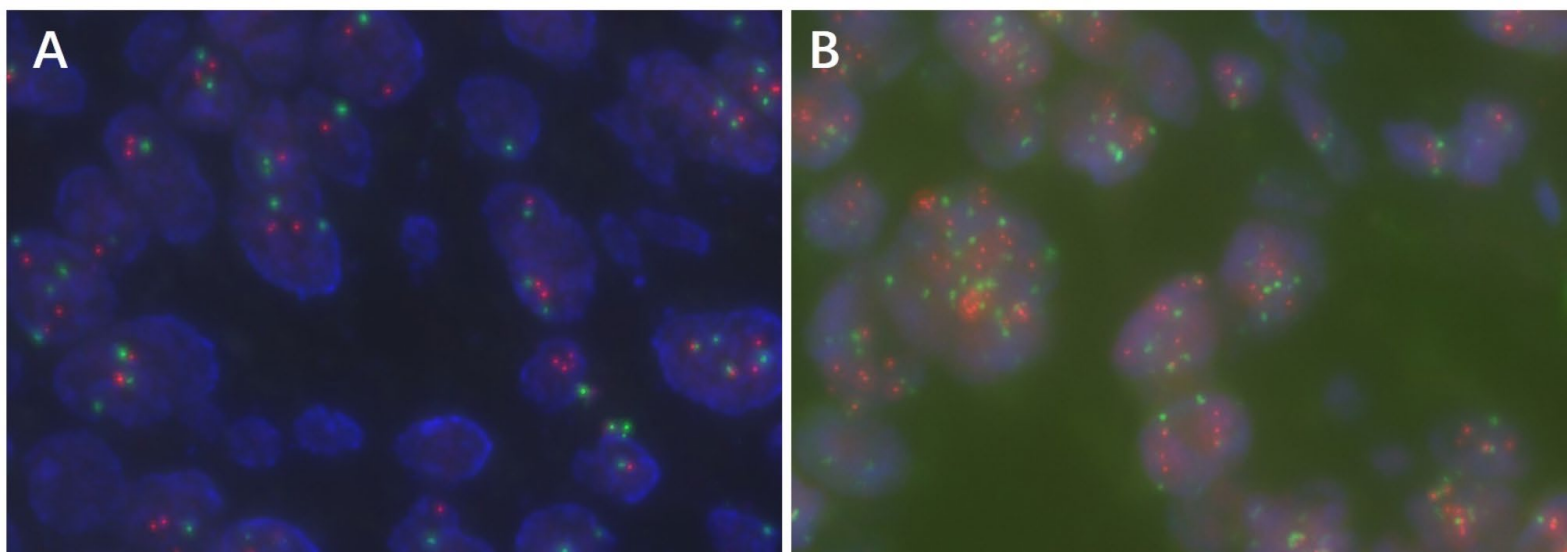
HER2 fluorescence in situ hybridisation staining in male breast cancer; **A**, HER2 FISH negative (no amplification); **B**, HER2 FISH positive (amplification)

**Fig. 5:**
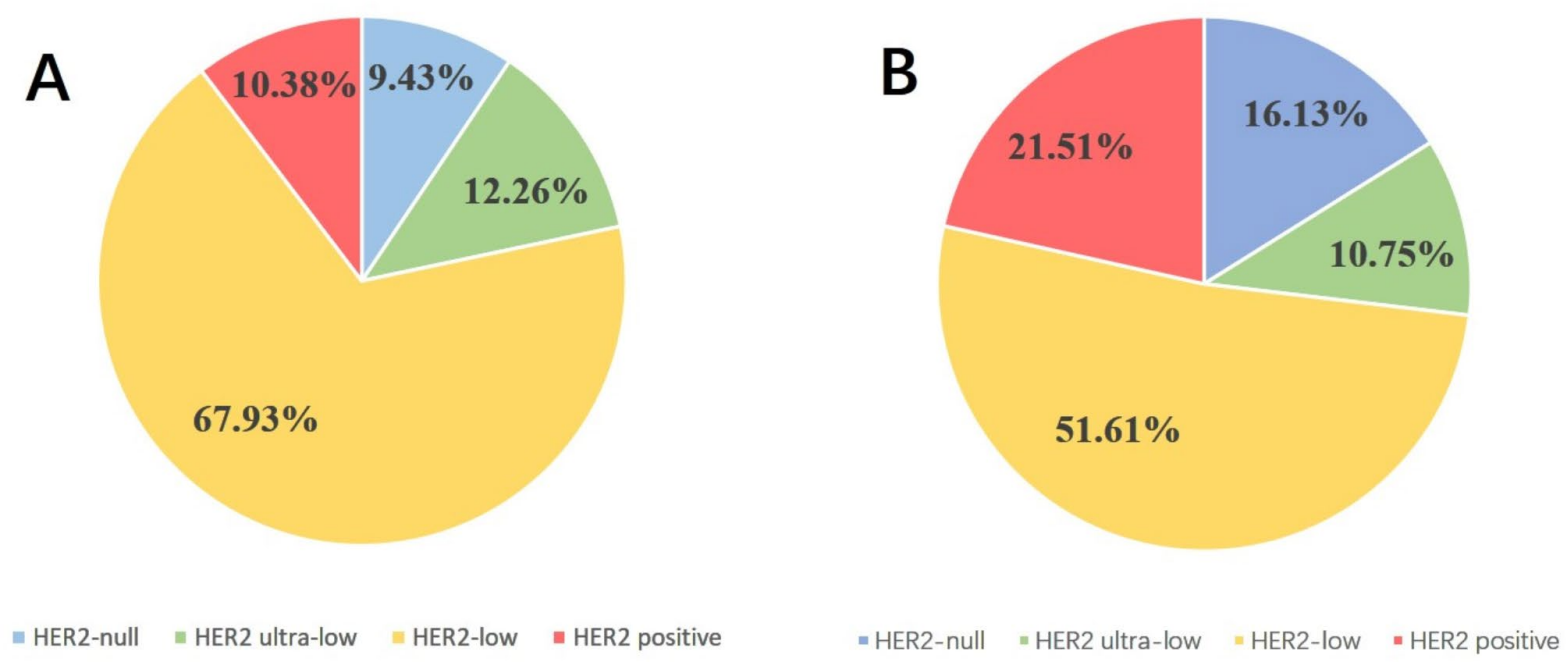
HER2 distribution of male breast cancer and female breast cancer; (**A**) male breast cancer, MBC; (**B**) female breast cancer, FBC

Patients with invasive breast cancer were enrolled for HER2 testing, and interpretation was based on the ASCO/CAP guidelines. HER2-negative cases were classified as HER2 low expression (IHC = 1+/2 + and no amplification by ISH) and HER2-0 (IHC-0, HER2 null and < 10% weak staining of cell membrane) (Figs. 3 and 4). Immunophenotype of male breast cancer: ER positive 99cases, accounting for 93.40%; PR positive 98cases, accounting for 92.45%; HER2 IHC 0 for 23 cases, HER2 IHC 1 + for 27 cases, HER2 IHC 2 + for 51 cases, HER2 IHC 3 + for 5 cases; HER2-null 10 cases, HER2 ultra-low 13 cases, HER2 low 72 cases, HER2-positive 11 cases, HER2-positive rate 10.38%, the incidence of low-expression 67.93%, the incidence of ultra-low expression 12.26%. Ki67 high-expression (≥ 14%) cases 87 cases, low-expression (< 14%) cases 19 cases. In the female breast cancers group, there were 25 cases of HER2 IHC 0, 28 cases of HER2 IHC 1+, 23 cases of HER2 IHC 2+, 17 cases of HER2 IHC 3+. There were 25 cases of HER2 zero, 48 cases of HER2 low, 20 cases of HER2 positive. 21.51% of HER2 positivity and 51.61% of low expression incidence. By statistical chi-square test analysis (χ2 =6.675 ), there was a difference in HER2 expression between male and female breast cancers, and the difference was statistically significant (*P* < 0.05) (Fig. 5).

### Prognosis analysis of male breast cancer

One hundred and six male patients with invasive breast cancer were included, of whom 96 had complete follow-up data, including 84 cases of modified radical surgery and 12 cases of simple lumpectomy. The follow-up period ended in June 2024 and ranged from 8 to 94 months, with a median follow-up period of 42 months and a loss-to-follow-up rate of 9.43%. Disease free survival (DFS) was defined as the time between surgery and the date of a breast cancer-related event, the date of last follow-up, or the date of death. Breast cancer-related events included local recurrence, distant metastasis, or death from breast cancer. Overall survival (OS) was defined as the total time from the first diagnosis of breast cancer to the patient’s death. 96 male breast cancer patients survived 91 and died in 5 cases by the cut-off date for follow-up. The DFS and OS were 85.42% and 94.79%, respectively.

**Fig. 6:**
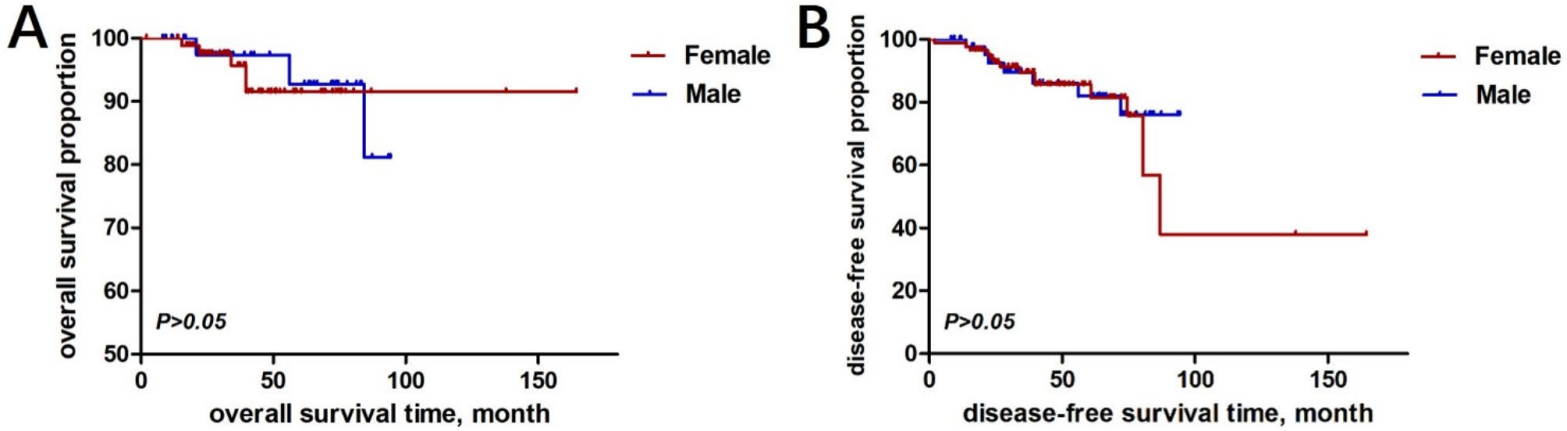
Prognostic survival analysis of male breast cancer versus female breast cancer; (**A**) overall survival, OS; (**B**) disease free survival, DFS

Ninety-three female invasive breast cancer patients were included in the control group, 88 of whom had complete follow-up data and underwent modified radical surgery. Follow-up cut-off date was June 2024 and ranged from 15 to 164 months, with a median follow-up time of 42 months and a loss-to-follow-up rate of 5.38%. Eighty-three of the 88 female breast cancer patients survived and five died by the cut-off date of follow-up. The DFS and OS were 84.09% and 94.32%. The Kaplan-Meier survival curves showed that there was no difference between male breast cancer and female breast cancer in terms of OS and DFS (*P* = 0.777 > 0.05; *P* = 0.668 > 0.05) (Fig. 6).

**Fig. 7:**
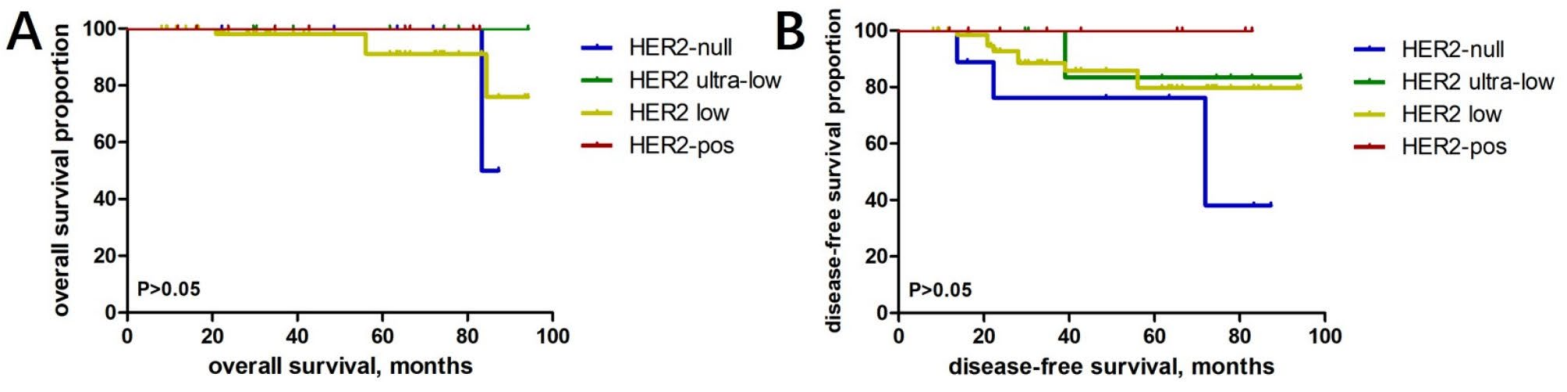
Survival analysis of different HER2 status groups of male breast cancer; **A** overall survival, OS; **B** disease-free survival, DFS

Prognostic analysis was performed according to the HER2 expression status of male breast cancer: 9 cases in the HER2-null group, 10 cases in the HER2 ultra-low group, 65 cases in the HER2 low group, and 10 cases in the HER2-positive group. The Kaplan-Meier survival curves of different subgroups showed that there was no difference in OS and DFS between different HER2 expression statuses (*P* = 0.758 > 0.05) (Fig. 7).

## Discussion

Male breast cancer (MBC), a rare type of breast cancer, may have a unique biology, and studies have shown a different distribution of intrinsic subtypes in MBC compared to female breast cancer, with a predominance of tubular A and B subtypes [11–12]. Men usually develop breast cancer at an older age compared to women. Histologically, MBC is usually grade 2, hormone receptor positive and HER2 negative. Reporting and staging was similar to that of female breast cancer [13].

The incidence of hormone receptor positivity was higher in MBC patients than in the hormone receptor negative group. Relevant studies have shown [14] that the vast majority of male breast cancer patients are of HR-positive/HER2-negative subtype (84.1%), followed by HR-positive/HER2-positive subtype (12.7%), while triple-negative breast cancers are uncommon (2.3%), and HR-negative/HER2-positive subtype is rarer (0.8%). In this study, the ER and PR positivity rates of male breast cancer could be over 90%, respectively; the HER2 positivity rate was 10.38%, the incidence of low-expression was 67.93%, and the incidence of ultra-low expression was 12.26%. This is basically consistent with previous reports, and its HER2 positivity rate is significantly lower than that of female breast cancer patients. Notably, the rate of HER2 low in FBC is significantly lower than what we observed in MBC [15].

The low and ultra-low HER2 expression status in breast cancer has attracted great attention with the emergence of new ADC analogues. T-DXd is an ADC drug that combines an anti-HER2 antibody with a potent cytotoxic carrier. This drug is unique in that it can effectively target tumour cells expressing low levels of HER2. However, there is still a gap regarding the characterisation of low HER2 expression in the male breast cancer population. Not only in terms of low HER2 expression, many previous studies have specifically excluded men with breast cancer due to the rarity of male breast cancer. A study by Duma et al. [16] evaluated 426 therapeutic breast cancer trials from January 2000 to April 2017 and found that 65% (*n* = 277) of the trials excluded men from the inclusion criteria. Treatment recommendations leading to male breast cancer are often extrapolated from the results of clinical trials that focus primarily on female breast cancer patients.

In this study, we investigated the clinicopathological features of HER2 low expression based on male breast cancer population, and also focused on the expression of HER2 ultra-low in male breast cancer, and the results showed that the incidence of HER2 low expression was significantly higher in male breast cancer than in female breast cancer. With the positive results of DESTINY-Breast 06 shown, the ADC class will cover a wider patient population. In the meantime, the inclusion of more male cases in future clinical studies is essential to ensure a full understanding of drug efficacy, safety, and potential gender response. More research is needed to determine whether male breast cancer is a better candidate for the new ADC class of drugs.

Numerous studies have explored the association between HER2 low expression and patient outcomes in FBC. Surveillance, Epidemiology, and End Results (SEER) data from 2005 to 2010 showed that men with breast cancer had a worse overall prognosis compared to women, with men having a lower 5-year survival rate than women patients (82.8% versus 88.5%), and a 43% higher risk of death than women after accounting for other variables [17]. A recent analysis [18] of SEER data from 2010 to 2017 showed that 2.3% of all male breast cancers were triple-negative, and in a multifactorial analysis, race, tumour type and stage were significantly associated with overall survival. In this study, the five-year DFS and OS for male breast cancer were 87.50% and 95.83%, respectively, whereas the five-year DFS and OS for female breast cancer in the control group were 87.50% and 94.32%, respectively, which did not show prognostic variability.

In the male breast cancer cohort, the different HER2 statuses were categorised as HER2-null, HER2-ultralow, HER2-low and HER2-positive, but there was no statistically significant difference between the four groups in terms of DFS and OS. In a large cohort study using National Cancer Database data in the US, Peifer et al. also found that HER2 low patients had a survival rate similar to those with HER2 zero tumors [19]. This was supported by other studies [20–22].

In addition, there are some limitations in the study, due to the lower incidence of male breast cancer, the included sample size is small, but the inclusion of female breast cancer in the same period as a control group better reflects the clinicopathological characteristics of HER2 low-expression male breast cancer. Of course, more follow-up studies are needed to verify the clinicopathological characteristics and prognosis of male breast cancer with low HER2 expression.

## Conclusion

Male invasive breast cancer is rarer and more common in older adults over 60 years of age, with high ER and PR positivity rates and high HER2 low expression rates. The higher HER2 low expression rate in male breast cancer may provide new anti-HER2 treatment decisions.

## Data Availability

The datasets used and analyzed in this study are available from the corresponding author upon reasonable request.
